# Public sentiments towards the use of *Wolbachia-Aedes* technology in Singapore

**DOI:** 10.1186/s12889-021-11380-w

**Published:** 2021-07-18

**Authors:** Christina Liew, Li Ting Soh, Irene Chen, Lee Ching Ng

**Affiliations:** 1 Environmental Health Institute, National Environment Agency, Singapore, Singapore; 2 School of Biological Sciences, Nanyang Technological University, Singapore, Singapore

**Keywords:** *Wolbachia*, Perception, Survey, Dengue, Singapore

## Abstract

**Background:**

*Wolbachia* technology is a novel vector control approach that can reduce mosquito populations and the risk of mosquito-borne diseases, which has recently gained popularity amongst countries. In 2016, Singapore embarked on a multi-phased field study named Project *Wolbachia –* Singapore, to evaluate the use of *Wolbachia* technology as an *Aedes aegypti* mosquito population suppression tool to fight dengue. Due to the novelty of this technology in Singapore, this study aims to understand the public’s acceptance and sentiments towards the use of *Wolbachia* technology.

**Methods:**

Several public sentiment survey approaches – including online, face-to-face in the streets, as well as door-to-door household surveys – were conducted.

**Results:**

The surveys conducted prior to the first field releases and implementation of the project revealed high support for the use of *Wolbachia* technology in Singapore. A household perception survey conducted in the interim of the first project phase was encouraging, with the majority of the respondents being aware of the project and having no concerns with the release of male *Wolbachia-*carrying *Aedes aegypti* (*Wolbachia-Aedes*) mosquitoes in their neighbourhood.

**Conclusions:**

The study reveal high support for the use of *Wolbachia* technology in Singapore and also provided invaluable insights that were used in the development of a public communications and engagement framework model, which thus helped to guide these elements in the subsequent phases and expansion of the project.

## Background

The use of *Wolbachia* strategy to suppress vector populations is a novel approach, which has the potential to reduce mosquito populations and the risk of mosquito-borne disease transmission [1, 2]. This approach, commonly known as the Incompatible Insect Technique (IIT), is a species-specific and benign approach for controlling vector populations [3]. Eggs produced from the successful mating between released male *Wolbachia-*carrying *Aedes aegypti* (*Wolbachia-Aedes*) mosquitoes and urban female *Aedes aegypti* mosquitoes in the environment (without *Wolbachia*) are non-viable, due to Cytoplasmic Incompatibility (CI), therefore suppression of mosquito populations could be achieved with regular releases over time [3, 4].

Field studies are necessary to demonstrate that the male *Wolbachia-Aedes* mosquitoes behave in the same way as they do in the laboratory, when subjected to environmental conditions in the field. The potential of using this strategy was first demonstrated in the suppression of *Culex pipiens* in Myanmar [5]. Improved technology in recent years made the microinjection of *Wolbachia* strains into mosquito species possible [6], allowing exploitation of the *Wolbachia* strategy for suppressing populations of mosquito species [7–9]. Numerous field trials have been reported recently, including successful population suppression of the Polynesian tiger mosquito, *Aedes polynesiensis*, in French Polynesia [10]; *Aedes albopictus* in China [11–13] and in the United States [14]; and *Aedes aegypti* in the United States [15], in Australia [16], as well as in Thailand [17]. To test the effectiveness of the *Wolbachia-Aedes* suppression approach, a multi-phased field study – named ‘Project *Wolbachia* – Singapore’ – was launched in 2016 by the National Environment Agency (NEA), a statutory board under the Ministry of Sustainability and the Environment (MSE) [previously known as the Ministry of the Environment and Water Resources, MEWR], Government of Singapore. This population suppression approach involves the release of male *Wolbachia-Aedes* mosquitoes in a high-rise and densely built urban environment.

Many such field trials acknowledge the complexities and challenges that come with politics and ethics, hence the importance of both community and government support are emphasised, in addition to high scientific quality [18]. With public consultations and engagement deemed critical for successful implementation of the *Wolbachia-Aedes* technology, several authors have recommended strategies for effective community engagement [18–22]. These strategies include understanding the socio-political context of the local population, close engagement with local communities informing them of the study, and periodic update of the project [16, 23].

In Singapore, extensive stakeholder engagement regarding the use of the *Wolbachia-Aedes* suppression approach was planned and put in place, prior to any possible field releases [24]. Early consultations with key relevant stakeholder groups – such as local and international academia, scientists, medical doctors, the local nature society, specific interest groups, community leaders, and residents at the selected study sites – allowed the identification of risks and understanding of community concerns, to help shape key public communications messaging and gain early support from these important groups. During project implementation, detailed information and timely updates were regularly provided through various channels, including: publicity banners and posters placed at strategic locations with high footfall at the study sites; educational brochures prepared in Singapore’s four official languages (English, Mandarin, Malay and Tamil) distributed to every single household/ other premises within the study sites; and regular, detailed informational updates provided via mainstream (print and online) media, social media, as well as NEA’s Project *Wolbachia* – Singapore webpage. Besides the dissemination of information, Learning Journeys to NEA’s Mosquito Production Facility, sharing sessions at schools and childcare centres, and mass outreach sessions via roadshows for the general public, were regularly conducted face-to-face, to generate awareness and understanding. Another key aspect of Singapore’s engagement strategy was ensuring a transparent and responsive feedback system to address concerns in the community – including via telephone hotline, email, an online reporting system, and verbal feedback to NEA field officers on the ground – which were crucial for maintaining trust with, and accountability to, our stakeholders.

Enhanced community engagement for the understanding of *Wolbachia* technology does not necessarily equate to public approval and acceptance. In several field studies, an individual informed-consent approach was adopted, and releases were done only at sites where authorisation was obtained from households in the community [16, 25]. While this approach is plausible for a small-scale field study, it is challenging for large-scale deployment [26].

To understand public acceptance and sentiments towards the use of *Wolbachia-Aedes* technology in Singapore, we adopted several public sentiment survey approaches, including online, face-to-face in streets, as well as door-to-door household surveys. Herein, we examined the public’s awareness of dengue, and the public’s opinion towards the use of *Wolbachia-Aedes* technology prior to the first field releases conducted in October 2016, as well as the perception of residents at the study sites in the interim of the first project phase. The association of social-demographic characteristics, knowledge and attitude of the public towards the release of male *Wolbachia-Aedes* were analysed. The surveys conducted also served as one of the channels for accountability to residents and stakeholders, gathering feedback from the community, and addressing residents’ concerns.

## Methods

### Online public perception survey

An independent consultancy firm was engaged to conduct an online public perception survey, to gather public perceptions and sentiments towards Project *Wolbachia* – Singapore, as part of our risk assessment on the use of *Wolbachia* technology. An email with an embedded link to the online survey portal was sent to a database of survey respondents, and the online survey portal was opened for three weeks from May to June 2016.

### Face-to-face street survey

A quick face-to-face street survey targeting the older population (aged above 40) was conducted over one weekend in August 2016, to complement the online survey results. The survey questionnaire captured demographic variables, such as age, gender, highest education level, and the respondents’ knowledge on and sentiments towards the use of *Wolbachia* technology. The survey was conducted using the QuestionPro web-based platform (QuestionPro.com). Surveyors were trained before conducting the surveys, to ensure that the objectives, methodology, expectations and questionnaires were well understood.

### Household perception survey

Household perception surveys were administered at all three selected release sites, at Braddell Heights, (Release site 1_BH), Tampines West (Release site 2_TW) and Nee Soon East (Release site 3_NSE), during the first two months of the small-scale field study, carried out from November to December 2016. The survey was conducted using the same structured questionnaire that was used in the face-to-face street survey interviews. Surveyors were trained before conducting the surveys, to ensure that the objectives, methodology, expectations and questionnaires were well understood.

#### Household perception survey at release site 1_BH

Release site 1_BH was a landed estate with private residential houses. Using the location map of the estate, surveyors approached residents of all the residential houses to conduct the household perception survey. One respondent from each household was interviewed, after obtaining his/her informed consent to participate. In the event of any of the following scenarios: (i) no respondent present at the household; (ii) respondent refused to participate; or (iii) unable to communicate with the respondent, the surveyor noted the response and proceeded to the next household in a systematic manner. Household responses that were recorded under scenario (i) on the first occasion were re-visited (up to two times) on different days and timings, in order to try obtaining a response. A total of 113 perception survey were collected at Release site 1_BH.

#### Household perception survey at release site 2_TW and release site 3_NSE

Release site 2_TW and Release site 3_NSE were residential estates with blocks of public housing flats, managed by the Housing & Development Board (HDB) of Singapore. Based on the household estimates at Release site 2_TW and Release site 3_NSE, the minimum survey sample size was calculated using Epi Info™ version 7.2 (Centers for Disease Control, Atlanta, US), using the following parameters: 5% confidence limits and 95% confidence level as shown in Table 1. The expected frequency of the outcome was considered at 50%, because the study covers several potential variables. Based on the minimum survey sample size, a representative number of surveys was conducted at each residential block at the study sites. A randomised household list was generated for each block, and door-to-door surveys were conducted for the randomised household list. One respondent from each household was interviewed, after obtaining his/her informed consent to participate. In the event of any of the following scenarios: i) no respondent present at the household; ii) respondent refused to participate; or iii) unable to communicate with the respondent, the next household in the list was invited to participate, until the minimum sample size required was reached.

**Table 1:** Sample size for survey conducted in Release site 2_TW and Release site 3_NSE

Selected sites	Household estimates	Survey sample size (minimum sample size)
Release site 2_TW	2941	382 (340)
Release site 3_NSE	1000	281 (278)

## Results

### Online public perception survey

A total of 1012 respondents participated in the online public perception survey. Comparing the survey population with the Singapore population figures in 2015, reflected in the Yearbook of Statistics 2016 [27] and the General Household Survey of 2015 [28], the survey results obtained over-represented younger and more educated respondents. As such, the analysis for respondents aged 40 years and above were excluded in this section, and the analysis was focused on younger respondents who were representative of the population, aged from 20 to 39 years (within about 5% of the population, as measured in 2015). Various adjustments were made to allow for a comparison, including merging groups, assuming uniform distributions of age categories, and mapping definitions of variables between data sets. The demographic profile of survey respondents is summarised in Table 2.

**Table 2:** Demographic profile of survey respondents who participated in the online perception survey

Categories	Respondent Distribution (%)	Population % (*From the Yearbook of Statistics 2016 and the General Household Survey of 2015*)	Adjusted Respondent Distribution (%) *After exclusion of population > 40*	Adjusted Population %
*Gender*				
Male	44%	49%	44%	51%
Female	56%	51%	56%	49%
*Age (years)*				
≤18	0%	*	0%	*
18–24	22%	12%	29%	30%
25–29	18%	9%	24%	22%
30–34	20%	9%	26%	24%
35–39	15%	10%	20%	25%
40–44	11%	10%	0%	*
45–55	11%	22%	0%	*
>55	3%	30%	0%	*
*Education Level*				
PSLE or none	1%	19%	1%	2%
‘O’-Levels or equivalent	12%	8%	10%	14%
‘A’-Levels or equivalent	7%	18%	7%	13%
Diploma	27%	27%	28%	18%
Degree or further	53%	27%	54%	53%

* Excluded for comparison; sum for other ages scaled to 100%

Survey responses were analysed, and the questions on: i) how comfortable participants are with the use of *Wolbachia* technology at a dengue hotspot near their homes; ii) how confident they are with *Wolbachia* technology; and iii) if they think the Government should use *Wolbachia* technology, were found to be highly correlated (Chronbach’s alpha ≈0.76). Hence in this paper, we focused our analysis on whether the survey respondents thought the government should implement *Wolbachia* technology in relation to their demographic background.

In general, 97% of the population (aged from 18 to 39 years) did not oppose the implementation of *Wolbachia* technology in Singapore. Analysis of the results found strong agreement with the Government’s implementation of *Wolbachia* technology (18% strongly agreed, 51% agreed, and only 3% disagreed or strongly disagreed). This agreement on implementation of *Wolbachia* technology was fairly consistent across all demographic groups considered. No significant difference in the support for use of *Wolbachia* technology was found among respondents of different age groups. However, a significant difference was observed among respondents of different genders and educational background. Compared to female respondents, men were less hesitant about the new technology, and more men were in favour of it (*p* = 0.00187). Compared to respondents with tertiary education, respondents with primary school level or lower education background were more inclined to be neutral or to disagree with the implementation of *Wolbachia* technology (*p* = 0.02). However in this study, the number of respondents (aged from 18 to 39 years) with primary school or lower education background was relatively small (*n* = 5), therefore estimates on the support for use of *Wolbachia* technology from this group of respondents were not reliable.

Further analysis was also done to assess other attitudes or knowledge that may have contributed to the respondents’ attitudes towards the use of *Wolbachia* technology, in particular their impression of the severity of the dengue situation in Singapore, approval of the Government’s approach to control dengue, and general knowledge of dengue. Results of the analysis are summarised in Table 3.

**Table 3:** Analysis of respondents’ attitude or knowledge that may have contributed to their attitude towards the use of *Wolbachia* technology

Attitude/knowledge	Should *Wolbachia* technology be implemented?	Analysis of results
Impression of dengue situation		Significant, p = 0.003
Approval of Government’s efforts in controlling dengue		Significant, p = 0.003
Knowledge of dengue		Not significant

#### Impression of the severity of the dengue situation in Singapore

Respondents’ impressions of the severity of the dengue situation in Singapore were categorised into three groups: (1) those who were unsure or did not feel that dengue is serious; (2) those who felt that dengue was serious but was under control; and (3) those who felt that dengue was serious and required action. A larger proportion of negative attitudes towards the use of *Wolbachia* technology was found among respondents in the category who expressed being unsure and generally did not feel that dengue is a serious problem (*p* = 0.003), compared to those in the other categories. However, it is noted that most individuals in this group were still supportive of the use of *Wolbachia* technology.

#### Approval of the Government’s approach to controlling dengue

Respondents’ responses were split into two categories: (1) those who approved, and (2) those who disapproved of the Government’s efforts in controlling dengue. Respondents who disagreed with the use of *Wolbachia* technology were mostly from category (2), who thought that the Government’s efforts were inadequate (*p* = 0.003).

#### Knowledge of dengue

Respondents were divided into three groups (high knowledge of dengue, moderate knowledge of dengue, and low knowledge of dengue), based on their answers to the following four dichotomous True/False questions about dengue infection.

**Table:** 

1. *Only female mosquitoes bite. Male mosquitoes do not bite.* 2. *Aedes aegypti, the main mosquito transmitter of dengue in Singapore, is more commonly found in urban areas than in forested areas.* 3. *There is currently no cure for dengue.* 4. *Dengue is only transmitted through mosquito bites.*	

No association was found between respondents’ knowledge of dengue and their support for the use of *Wolbachia* technology.

To better understand the areas of concern that the public may have on the use of *Wolbachia* technology, respondents were tasked to rank the top three highest concerns below:

**Table:** 

1. *Negative impact on the ecosystem* 2. *Increase in mosquitoes in the environment* 3. *Negative effects of* Wolbachia *on human health* 4. *Irritation/anxiety due to increase in mosquitoes* 5. *Doubts about the effectiveness of* Wolbachia *technology in controlling dengue* 6. *Unknown side effects of* Wolbachia *(*e.g. *mutation of mosquitoes)* 7. *Increase in my overall expenditure on controlling dengue* 8. *Government/authorities will have to spend more on controlling increased populations of mosquitoes*	

The top three concerns amongst the respondents were: the unknown side effects of *Wolbachia* (33%); unspecified negative impact on human health (26%); and impact on the ecosystem (25%). Additional costs (incurred by individuals or the Government) were rarely mentioned as a concern, regarding the use of *Wolbachia* technology.

In addition, respondents were asked about their possible reaction, following the hypothetical establishment of a *Wolbachia-Aedes* suppression programme. A significant proportion of respondents (81%) speculated that they would continue with their current efforts to prevent mosquito breeding. A small number of individuals stated that they would stop current efforts to prevent mosquito breeding.

### Face-to-face street survey

Two groups (those aged over 40 years, and those who had completed their education at primary or secondary school level) were substantially under-represented in the initial online survey. Therefore a face-to-face street survey targeting the older population (aged above 40) was conducted, to complement the online survey results. A total of 163 respondents participated in the face-to-face street survey, with the demography of the respondents summarised in Table 4.

**Table 4:** Demographic profiles of respondents in the face-to-face street survey

Categories	Respondent Distribution, *N* = 163 *(n* %)
*Gender*	
Male	83 (51%)
Female	80 (49%)
*Age (years)*	
≤ 20	0 (0%)
21–30	5 (3%)
31–40	4 (2%)
41–50	37 (23%)
51–60	52 (32%)
>60	65 (40%)
*Education Level*	
PSLE and below	41 (25%)
‘O’-Level or equivalent	37 (23%)
‘A’-Level or equivalent	7 (4%)
Diploma	22 (13%)
Degree	34 (31%)
Postgraduate degree (MSc/PhD)	22 (13%)

89% of the street survey respondents had no concern (they either agreed or were neutral) with the release of male *Wolbachia-Aedes* mosquitoes in their neighbourhood. In addition, 31% of the street survey respondents had heard of Project *Wolbachia* – Singapore.

### Household perception survey

A total of 776 household perception surveys were administered at all three selected release sites –Release site 1_BH (*N* = 113), Release site 2_TW (*N* = 382), and Release site 3_NSE(*N* = 281). As male *Wolbachia-Aedes* releases at Release site 1_BH were conducted at a single point in the landed estate while releases in Release site 2_TW and Release site 3_NSE public housing estates were conducted at multiple points on ground floors, analysis of Release site 1_BH was done separately from Release site 2_TW and Release site 3_NSE.

#### Release site 1_BH

At Release site 1_BH, 88% (*n* = 100) of the households surveyed had heard of Project *Wolbachia* – Singapore, and of the various outreach materials distributed and activities conducted, the majority of households first heard of the project through the news (32%), publicity materials (23%), door-to-door house visits conducted by project officers (20%), and during the garden party organised in the community prior to the first releases of male *Wolbachia-Aedes* mosquitoes (18%)*.* 88% of the households (*n* = 100) at Release site 1_BH, had no concerns with the release of male *Wolbachia-Aedes* mosquitoes in their neighbourhood. However, a small concentration of households near the release site (single point release) expressed that they were uncomfortable with the release.

The socio-demographic factors of respondents, their perception towards the Government’s efforts, and their awareness of the dengue situation, were then analysed, to identify an attributing factor that may contribute to a positive outcome (comfortable/very comfortable with male *Wolbachia-Aedes* release), as summarised in Table 5. Univariate analysis showed no association between the socio-demography of respondents and their acceptance towards male *Wolbachia-Aedes* release. Looking at the factors that may contribute to higher awareness of dengue (feels that dengue is a serious problem/serious problem but under control), the odds of a respondent with a diploma/degree/postgraduate degree (compared to having a Primary School Leaving Examination or PSLE certificate, and below) feeling that dengue is a serious problem/serious problem but under control was 5.14 times more likely.

**Table 5:** Analysis of socio-demographic factors of respondents at Release site 1_BH, their perception towards Government’s efforts, their awareness of the dengue situation, and their attitude towards *Wolbachia* release

	Positive attitude towards male ***Wolbachia-Aedes*** release (very comfortable/comfortable)				High awareness of dengue (feels that dengue is a serious problem/serious problem but under control)			
	N	n (%)	Odds Ratio [95% CI]	*p*-value	N	n (%)	Odds Ratio [95% CI]	*p*-value
**Gender**								
Male	52	40 (77%)	Reference		52	38 (73%)	Reference	
Female	61	39 (64%)	0.53 [0.35,0.81]	0.14	61	38 (62%)	0.61 [0.40, 0.92]	0.225
**Age (years)**								
≤35	20	15 (75%)	Reference		20	14 (70%)	Reference	
36–55	40	27 (68%)	0.69 [0.37,1.28]	0.55	40	33 (83%)	2.02 [1.06,3.84]	0.27
>55	53	37 (70%)	0.77 [0.42,1.40]	0.66	53	29 (55%)	0.52 [0.30,0.91]	0.24
**Education level**								
PSLE and below	15	11 (73%)	Reference		15	6 (40%)	Reference	
‘O’/ ‘A’ - Level or equivalent	36	23 (64%)	0.64 [0.33,1.27]	0.51	36	22 (61%)	2.36 [1.26,4.42]	0.17
Diploma/ Degree/ Postgraduate degree	62	45 (73%)	0.96 [0.50,1.84]	0.95	62	48 (77%)	5.14 [2.80,9.45]	**0.007**
**Perception towards Government’s efforts**								
Extremely inadequate/ Inadequate	12	7 (58%)	Reference		12	11 (92%)	Reference	
Neutral	11	8 (73%)	1.90 [0.78,4.66]	0.47	11	4 (36%)	0.052 [0.015,0.18]	0.015
Adequate	68	46 (68%)	1.49 [0.79,2.83]	0.53	68	48 (71%)	0.22 [0.074,0.64]	0.16
Extremely adequate	22	18 (82%)	3.21 [1.44,7.19]	0.15	22	13 (59%)	0.13 [0.042,0.41]	0.073
**Awareness of dengue situation**								
Not serious	22	13 (59%)	Reference					
Unsure	15	11 (73%)	1.90 [0.92,3.94]	0.38				
Serious	21	17 (81%)	2.94 [1.45,5.95]	0.13				
Serious but under control	55	38 (69%)	1.55 [0.92,2.61]	0.40				

#### Release site 2_TW and release site 3_NSE

The majority of the households surveyed at Release site 2_TW (69%) and Release site 3_NSE (72%) had heard of Project *Wolbachia* – Singapore, and of the various outreach materials distributed and activities conducted, most first heard of the project through the news (Release site 2_TW: 56%; Release site 3_NSE: 35%) and publicity materials (Release site 2_TW: 24%; Release site 3_NSE: 33%). At both sites, 92% of the households had no concerns with the release of male *Wolbachia-Aedes* mosquitoes in their neighbourhoods.

The socio-demographic factors of respondents from Release site 2_TW and Release site 3_NSE, their perception towards the Government’s efforts, and their awareness of the dengue situation, were analysed, to identify an attributing factor that may contribute to a positive outcome (comfortable/very comfortable with male *Wolbachia-Aedes* release), as summarised in Tables 6, 7 and 8.

**Table 6:** Analysis of socio-demographic factors of respondents at Release site 2_TW and Release site 3_NSE that attribute to positive attitude towards male *Wolbachia-Aedes* release

	Release site 2_TW				Release site 3_NSE			
	Positive attitude towards male ***Wolbachia-Aedes*** release (very comfortable/comfortable)				Positive attitude towards male ***Wolbachia-Aedes*** release (very comfortable/comfortable)			
	N	n (%)	Odds Ratio [95% CI]	p-value	N	n (%)	Odds Ratio [95% CI]	p-value
**Gender**								
Male	176	134 (76%)	Reference		109	90 (83%)	Reference	
Female	206	152 (74%)	0.88 [0.70,1.12]	0.60	172	131 (72%)	0.67 [0.49,0.91]	0.20
**Age (years)**								
≤35	127	86 (68%)	Reference		88	65 (74%)	Reference	
36–55	98	82 (84%)	2.44 [1.75,3.41]	**0.007**	86	70 (81%)	1.55 [1.07,2.24]	0.24
>55	157	118 (75%)	1.44 [1.11,1.88]	0.17	107	86 (80%)	1.45 [1.03,2.04]	0.28
**Education level**								
PSLE and below	139	100 (72%)	Reference		109	86 (79%)	Reference	
‘O’/ ‘A’ - Level or equivalent	132	108 (82%)	1.76 [1.31,2.36]	0.056	88	70 (80%)	1.04 [0.73,1.48]	0.91
Diploma/ Degree/ Postgraduate degree	111	78 (70%)	0.92 [0.70,1.22]	0.77	84	65 (77%)	0.91 [0.64,1.30]	0.80
**Perception towards Government’s efforts**								
Extremely inadequate/ Inadequate	25	15 (60%)	Reference		19	11 (58%)	Reference	
Neutral	53	31 (58%)	0.94 [0.57,1.54]	0.90	48	32 (67%)	1.45 [0.83,2.53]	0.50
Adequate	263	208 (79%)	2.52 [1.63,3.90]	**0.033**	172	144 (84%)	3.74 [2.25,6.22]	**0.009**
Extremely adequate	41	32 (78%)	2.37 [1.36,4.13]	0.12	42	34 (81%)	3.09 [1.68,5.68]	0.06
**Awareness of dengue situation**								
Not serious	46	38 (83%)	Reference		36	31 (86%)	Reference	
Unsure	41	24 (59%)	0.30 [0.18,0.49]	0.016	27	20 (74%)	0.46 [0.24,0.88]	0.23
Serious	77	54 (70%)	0.49 [0.31,0.78]	0.13	62	44 (71%)	0.39 [0.23, 0.69]	0.09
Serious but under control	218	170 (78%)	0.75 [0.49,1.14]	0.49	156	126 (81%)	0.68 [0.40,1.14]	0.46

**Table 7:** Multinomial regression analysis to compare respondents’ acceptance towards male *Wolbachia-Aedes* release and their perception of the Government’s efforts in controlling dengue

**Release site 2_TW**		**Perception of Government’s efforts in controlling dengue**			
		**Extremely inadequate/ inadequate** **(Reference)**	Neutral* **OR (*p)***	**Adequate OR (*p)***	**Extremely adequate** **OR (*p)***
**Acceptance towards male** ***Wolbachia-Aedes*** **release**	Very uncomfortable/ uncomfortable	**6.74 (0.02)**		2.01 (0.26)	2.25 (0.37)
	Neutral (Reference)				
	Comfortable	2.41 (0.17)		**3.35 (0.0006)**	2.14 (0.17)
	Very comfortable	0.0004 (0.95)		1.58 (0.52)	**12.0 (0.002)**
**Release site 3_NSE**		**Perception of Government’s efforts in controlling dengue**			
		**Extremely inadequate/inadequate** **(Reference)**	Neutral* **OR (*p*)**	**Adequate** **OR (*p*)**	**Extremely adequate** **OR (*p*)**
**Acceptance towards male** ***Wolbachia-Aedes r*elease**	Very uncomfortable/ uncomfortable	**9.0 (0.03)**		1.42 (0.62)	3.0 (0.23)
	Neutral (Reference)				
	Comfortable	1.74 (0.52)		**2.79 (0.01)**	2.90 (0.09)
	Very comfortable	12.0 (0.08)		4.42 (0.18)	**12.0 (0.048)**

*“Neutral” is taken as reference for acceptance towards male *Wolbachia-Aedes* release and perception of the Government’s efforts

**Table 8:** Analysis of socio-demographic factors of respondents at Release site 2_TW and Release site 3_NSE that attribute to high awareness of the dengue situation

	Release site 2_TW				Release site 3_NSE			
	High awareness of dengue (feels that dengue is a serious problem/serious problem but under control)				High awareness of dengue (feels that dengue is a serious problem/serious problem but under control)			
	N	Odds Ratio [95% CI]	Odds Ratio [95% CI]	p-value	N	Odds Ratio [95% CI]	Odds Ratio [95% CI]	p-value
**Gender**								
Male	176	140 (80%)	Reference		109	79 (72%)	Reference	
Female	206	155 (75%)	1.20 [0.87,1.64]	0.57	172	139 (81%)	1.69 [1.18,2.42]	0.14
**Age (years)**								
≤ 35	127	111 (87%)	Reference		88	73 (83%)	Reference	
36–55	98	84 (86%)	2.00 [1.09,3.69]	0.25	86	71 (83%)	1.13 [0.66,1.94]	0.82
> 55	157	100 (64%)	0.33 [0.23,0.49]	0.004	107	74 (69%)	0.41 [0.26,0.64]	**0.04**
**Education level**								
PSLE and below	139	92 (66%)	Reference		109	72 (66%)	Reference	
‘O’/ ‘A’ - Level or equivalent	132	104 (79%)	1.67 [1.18,2.35]	0.14	88	80 (91%)	4.20 [2.50,7.05]	**0.006**
Diploma/ Degree/ Postgraduate degree	111	99 (89%)	6.15 [3.54,10.7]	**0.001**	84	66 (79%)	2.11 [1.38,3.23]	0.08
**Perception towards Government’s efforts**								
Extremely inadequate/ Inadequate	25	18 (72%)	Reference		19	15 (79%)	Reference	
Neutral	53	34 (64%)	0.90 [0.43,1.87]	0.88	48	32 (67%)	2.29 [1.10,4.78]	0.26
Adequate	263	211 (80%)	1.10 [0.58,2.10]	0.88	172	136 (79%)	2.02 [1.10,3.73]	0.25
Extremely adequate	41	32 (78%)	0.66 [0.32,1.39]	0.58	42	35 (83%)	1.33 [0.66,2.68]	0.68

Using univariate analysis, the middle-age group (OR = 2.44, 95% CI = 1.75–3.41; *p* = 0.007) was found to be associated with a positive attitude towards male *Wolbachia-Aedes* release at Release site 2_TW. However, a further Chi squared test showed no significant correlation (*p* = 0.063).

Respondents with the perception of the Government’s efforts in controlling dengue being adequate at both Release site 2_TW (OR = 2.37, 95% CI = 1.63–3.90; *p* = 0.033) and Release site 3_NSE (OR = 3.74, 95% CI = 2.25–6.22; *p* = 0.009) were also found to be associated with a positive attitude towards male *Wolbachia-Aedes* release. A further Chi squared test done showed significant correlation between the impression of adequate Government efforts and a positive attitude towards male *Wolbachia-Aedes* release for both sites – Release site 2_TW (*p* = 4.91 × 10^− 10^) and Release site 3_NSE (*p* = 0.0009).

Further multinomial regression analysis performed found that the odds of a respondent who felt that the Government’s efforts were extremely adequate was 12 times more likely to be very comfortable towards male *Wolbachia-Aedes* release, as shown in Table 7).

Using univariate analysis, the older age group (OR = 0.41, 95% CI = 0.26–0.64; *p* = 0.04) was found to be associated with a high awareness of dengue (feels that dengue is a serious problem/serious problem but under control) at Release site 3_NSE. However, a further Chi squared test showed no significant correlation (*p* = 0.06).

Respondents at Release site 2_TW who had at least a diploma tended to feel that dengue was a serious problem/serious problem but under control (OR = 6.15, 95% CI = 3.54–10.7; *p* = 0.001); a further Chi squared test done showed significant correlation (*p* = 0.0003). At Release site 3_NSE, respondents with ‘O’/‘A’ Level qualifications tended to feel that dengue was a serious problem/serious problem but under control (OR = 4.30, 95% CI = 2.50–7.05; *p* = 0.006); a further Chi squared test done showed significant correlation (*p* = 1.99 × 10^− 5^).

Taking the survey response “dengue is not serious” as the reference point for dengue awareness, and “PSLE and below” as the reference point for education level, the respondent’s education level and their awareness of dengue was compared using multinomial regression analysis. In general, respondents at both release sites with at least ‘O’/‘A’ Level qualifications tended to feel that dengue was a serious problem/serious problem but under control. The results are summarised in Table 9.

**Table 9:** Multinomial regression analysis to compare respondents’ education level and their awareness of dengue

**Release site 2_TW**		**Awareness of dengue**			
		**Not serious** **(Reference)**	**Unsure** **OR (*p)***	**Serious** **OR (*p)***	**Serious but under control OR (*p)***
**Education Level**	PSLE and below (Reference)				
	‘O’ or ‘A’ Level		0.93 (0.88)	1.21 (0.64)	**2.17 (0.032)**
	Diploma/Degree/Postgraduate		2.48 (0.18)	**4.26 (0.017)**	**8.45 (0.00015)**
**Release site 3_NSE**		**Awareness of dengue**			
		**Not serious** **(Reference)**	**Unsure** **OR (*p*)**	**Serious** **OR (*p*)**	**Serious but under control OR (*p*)**
**Education Level**	PSLE and below (Reference)				
	‘O’ or ‘A’ Level		0.88 (0.87)	**3.12 (0.045)**	**6.22 (0.0006)**
	Diploma/Degree/Postgraduate		1.47 (0.51)	0.71 (0.53)	**3.40 (0.006)**

“Not serious” is taken as the reference for dengue awareness among respondents. “PSLE and below” is taken as the reference for education level of respondents

## Discussion

Understanding the public’s concerns and sentiments provided invaluable insights on how to better engage residents and stakeholders in the community, to generate awareness and understanding of the *Wolbachia* technology, and address any feedback and concerns. Results from the online survey conducted before the first releases and trialling implementation of the *Wolbachia* technology in the small-scale field study, suggested that a small proportion of individuals may feel complacent and stop trying to prevent mosquito breeding with the implementation of *Wolbachia* technology [29, 30]. This led us to highlight the importance of continued individual efforts in preventing mosquito breeding as one of the key messages, during extensive community engagement efforts at the study sites before the first releases and small-scale field study began.

From the survey data, we gleaned insights on the awareness and attitudes towards dengue among the online respondents in Singapore, as well as from the residents living at specific study sites. About 88% of the online survey respondents, and 68 to 77% of the residents at the selected study sites (BH, TW and NSE), perceived dengue as a serious problem in Singapore. 73% of the online survey respondents, and about 66 to 80% of the residents at the selected study sites, felt that the Government’s efforts to control dengue in Singapore were adequate. Studies done in other countries among urban populations revealed similar high levels of agreement that dengue is a serious disease, such as in Yemen (97.7%) and India (89%) [31, 32]. In the current study, social-demography, such as education level, had an influence in the level of awareness of dengue among the respondents. Therefore, easy-to-remember educational campaigns on dengue prevention would be most beneficial in reaching out to this small group of the population who are less educated, narrowing the knowledge gap in the awareness of dengue. Good knowledge on the risk of dengue would contribute towards greater motivation to carry out dengue control measures at the individual and community levels, as well as for greater support towards the Government’s efforts, such as using *Wolbachia* technology, in controlling dengue. In addition, educational campaigns leveraging the fear element would only be effective if the perceived threat is strong [33].

In this paper, we describe a high level of receptivity towards the use of *Wolbachia-Aedes* technology as a promising control tool to suppress the *Aedes aegypti* mosquito vector population in Singapore, gleaned from the various public sentiment surveys conducted by the team. This high level of receptivity may be attributed to a high level of trust felt by the general public towards the Government of Singapore. In a recent study conducted by the Institute of Policy Studies (IPS) Social Lab, a high level of confidence in the Government of Singapore was reported, ranking second highest amongst studies conducted in 12 other countries [34]. Particularly in the area of handling public health, it was reported that Singaporeans place a lot of trust in, and are satisfied with, the Government of Singapore, despite there sometimes being low levels of knowledge in this area amongst the respondents [35].

Sixty-nine to 88% of the respondents interviewed in the study had heard of Project *Wolbachia* – Singapore, with the majority of respondents (32 to 56%) hearing of the project through mainstream media (print and broadcast media) and publicity materials (23 to 33%). Similar findings have also been reported elsewhere, where mass media such as television and newspaper were identified as the major sources of public health information [36, 37]. However, a survey done in Singapore showed that digital news and social media platforms are growing in popularity due to the convenience of mobile devices, with majority relying on digital platforms of key news media for quick, timely and accurate information [38]. As such, updates on the project at various key milestones continued to be provided via various media channels, including mainstream media, to keep residents well informed about the progress of the project. Information was also regularly made available on the NEA webpage, as well as via digital and social media platforms.

The findings of the surveys reported here should be cautiously interpreted for several reasons. Although the online survey was intended for gathering public perceptions and sentiments towards Project *Wolbachia* – Singapore, the data gathered was skewed towards the younger population. A face-to-face street survey was conducted to complement the online survey, but the small sample size may have limited the precision to draw any accurate correlation.

Another limitation to consider was administering of the household perception survey, which was done during working hours, when many residents would have been at work and thus away from their homes. Thus the household survey done at the study sites may have been biased towards residents who were present at home during the day, such as homemakers. Nevertheless, the persons interviewed could actually have most adequately represented the general sentiments of the public towards the release of male *Wolbachia-Aedes* mosquitoes, as they would have been the ones most likely experiencing the increase in number of male *Wolbachia-Aedes* mosquitoes around their neighbourhoods, since the mosquito releases were carried out during the day.

As the project progressed, a subsequent survey was planned, to explore sentiments in greater detail, and to understand the knowledge, attitudes and outcomes using implicit question types. This would provide an understanding of whether there were any changes to the support from residents after two years of project implementation, and would provide insights on any difference in perception of the project at newer areas to which the project was expanded. Understanding of community perception would help guide subsequent educational campaigns and publicity.

## Conclusions

The various surveys conducted provided a good understanding of the public acceptance and sentiments towards the use of *Wolbachia-Aedes* technology in Singapore. The majority of residents at the study sites had heard of the ongoing Project *Wolbachia* – Singapore, involving the novel approach of releasing male *Wolbachia-Aedes* mosquitoes, which was a testament to the project team’s efforts in putting out extensive public communications and intensely engaging the community via a multitude of platforms. The surveys also revealed that the majority of the Singapore population recognises the seriousness of dengue, although a small proportion of the community does not perceive the high risk of dengue, which suggests the need for educational campaigns on the risk of dengue to be more visual, less wordy, and easier to remember. As the project scales up in subsequent phases, there is a need for longitudinal studies to understand how residents’ perception and behaviour change over time, and also for in-depth surveys to better identify gaps in the knowledge and practices related to the prevention of dengue.

## Data Availability

The datasets used and/or analysed during the current study are available from the corresponding author on reasonable request.

## Abbreviations

BH: Braddell Heights
CI: confidence interval
NEA: National Environment Agency
OR: odds ratio
PSLE: Primary School Leaving Examination
TW: Tampines West
NSE: Nee Soon East
